# Multiphase CT-Based Tumor and Peritumoral Radiomics for Characterization of Clear Cell Renal Cell Carcinoma

**DOI:** 10.1007/s10278-025-01797-9

**Published:** 2026-01-05

**Authors:** Matt Muellner, S. J. Pawan, Xiaomeng Lei, Manju Aron, Mihir Desai, Neha Gurram, Mansi Garg, Steven Y. Cen, Vinay Duddalwar

**Affiliations:** 1 Radiomics Lab, Department of Radiology, University of Southern California, Los Angeles, CA 90033 USA; 2 Department of Radiology, Keck School of Medicine, University of Southern California, Los Angeles, CA 90033 USA; 3 Department of Pathology, Keck School of Medicine, University of Southern California, Los Angeles, CA 90033 USA; 4 Department of Radiology, Los Angeles General Medical Center, Los Angeles, CA 90033 USA; 5 Institute of Urology, University of Southern California, Los Angeles, CA 90033 USA; 6 Alfred E Mann Department of Biomedical Engineering, USC Viterbi School of Engineering, Los Angeles, CA 90089 USA

**Keywords:** Clear cell renal cell carcinoma, Radiomics, Multiphase CT, Peritumoral region, Tumor characterization, Machine learning

## Abstract

Clear cell renal cell carcinoma (ccRCC) prognosis is guided by a tumor’s pathological features. While radiomics shows promise for non-invasive tumor characterization, it remains unclear whether incorporating the peritumoral region and leveraging multiphase CT imaging improves model performance beyond standard tumor-region analysis. This study aimed to determine whether radiomic features derived from the peritumoral area in standard four-phase CT protocol improve the prediction of ccRCC stage, grade, and aggressiveness compared to intratumoral features alone. A retrospective cohort of 250 patients with ccRCC was analyzed. Using tumor and 5-mm peritumoral regions on four-phase CT, 1874 radiomic features were extracted per region per phase. Elastic Net and Random Forest models were trained with tenfold cross-validation to predict binarized stage, grade, and aggressiveness outcomes using three feature sets: tumor region, peritumoral region, and a combined region including both the tumor and peritumoral regions. Models using only tumor-region features consistently outperformed those using the peritumoral and combined regions. The best tumor-region model achieved area under the receiver operating characteristic curve (AUC) values of 0.875 for stage, 0.697 for grade, and 0.915 for aggressiveness. Multiphase analysis provided no benefit over a single-phase approach, with the nephrographic phase alone yielding equivalent or superior performance to multiphase models across all outcomes. A simplified radiomic approach using features from the tumor region in the nephrographic phase alone provides optimal performance for predicting key ccRCC characteristics. The added complexity of peritumoral and multiphase analysis did not enhance predictive accuracy, potentially streamlining future ccRCC radiomic research.

**Supplementary Information:**

The online version contains supplementary material available at https://doi.org/10.1007/s10278-025-01797-9.

## Background

Renal cell carcinoma (RCC) accounts for over 90% of kidney cancers and more than 2% of all new malignancies diagnosed in adults [1, 2]. The clear cell subtype (ccRCC), most commonly arising from epithelial cells of the proximal tubule of the nephron, constitutes approximately 80% of these cases [3–5]. Factors such as pathological stage and grade are critical determinants of prognosis and therapeutic strategy [4, 6]. Contrast-enhanced multiphase computed tomography (CT) is the primary imaging modality for ccRCC, providing a non-invasive means for tumor detection, staging, treatment monitoring, and surgical planning [7]. Beyond qualitative assessment, CT images contain an abundance of high-dimensional quantitative data that can be leveraged through computational analysis to support clinical decision-making [8–10].

Radiomics, the extraction and analysis of quantitative features from medical images, has emerged as a promising tool for cancer characterization [11, 12]. Within the field of RCC, radiomic analysis of the primary tumor region has showcased potential in the prediction of tumor stage, grade, recurrence, and post-resection survival [13–17]. Furthermore, tumor-derived radiomic features can complement traditional clinical and pathological data by predicting biomarkers of treatment response [18, 19]. These developments position radiomics as a valuable non-invasive technology for refining personalized treatment strategies for patients with RCC.

Despite progress in RCC outcome prediction, much of the previous literature in RCC has focused exclusively on the primary tumor space [13, 20, 21]. Growing evidence across multiple cancer types indicates that the peritumoral region, comprised of the tissue immediately surrounding the tumor, holds significant prognostic value [22–24]. This region may capture critical tumor-host interactions that are important to tumor behavior, such as angiogenesis and inflammatory infiltration, which are not fully represented within the core tumor area [25–27]. Within the field of ccRCC, studies by Zhou et al. and Li et al. have demonstrated peritumoral radiomic features to improve the prediction of tumor grade, outperforming models based on features from the tumor region alone [27, 28].

A key limitation of prior peritumoral radiomics research in RCC is its reliance on single-phase CT imaging [17, 28, 29]. The multiphase protocol routinely used for ccRCC characterization captures distinct hemodynamic and structural properties of the tumor and its microenvironment across different contrast phases. The corticomedullary phase is rich in vascular kinetics and may best reveal peritumoral angiogenesis and blood supply [30]. The nephrographic phase, which best captures parenchymal contrast, could highlight infiltrative patterns and hypovascular abnormalities that are less apparent in other phases [31]. The excretory phase may be useful in evaluating washout dynamics [30, 31]. The potential for these multiphase data to provide complementary information from the peritumoral region has been largely unexplored.

This study aims to determine whether incorporating peritumoral radiomic features derived from four-phase contrast-enhanced CT improves the prediction of ccRCC stage, grade, and aggressiveness beyond intratumoral features alone. We hypothesize that a combined model leveraging both tumor and peritumoral features across multiple phases will outperform models based solely on the tumor region.

The main contributions of this work are:

- A comprehensive evaluation of peritumoral radiomics for predicting three clinical endpoints (stage, grade, and aggressiveness) in ccRCC.
- The integration of a standardized, four-phase CT protocol into peritumoral radiomic analysis to assess its added value, with comparative analysis of multiphase and single-phase modeling to determine the most efficient radiomic strategy for clinical prediction.

The remainder of this paper is organized as follows. The “Methods” section details our patient cohort and methodology. The “Results” section presents the performance of our models in predicting ccRCC stage, grade, and aggressiveness, comparing tumor, peritumoral, and combined region feature sets across single-phase and multiphase CT. The “Discussion” section explores the clinical implications of these findings, as well as comparing them with the prior literature. The “Conclusion” summarizes our key findings and their significance for future research.

## Methods

### Study Design

**Fig. 1:**
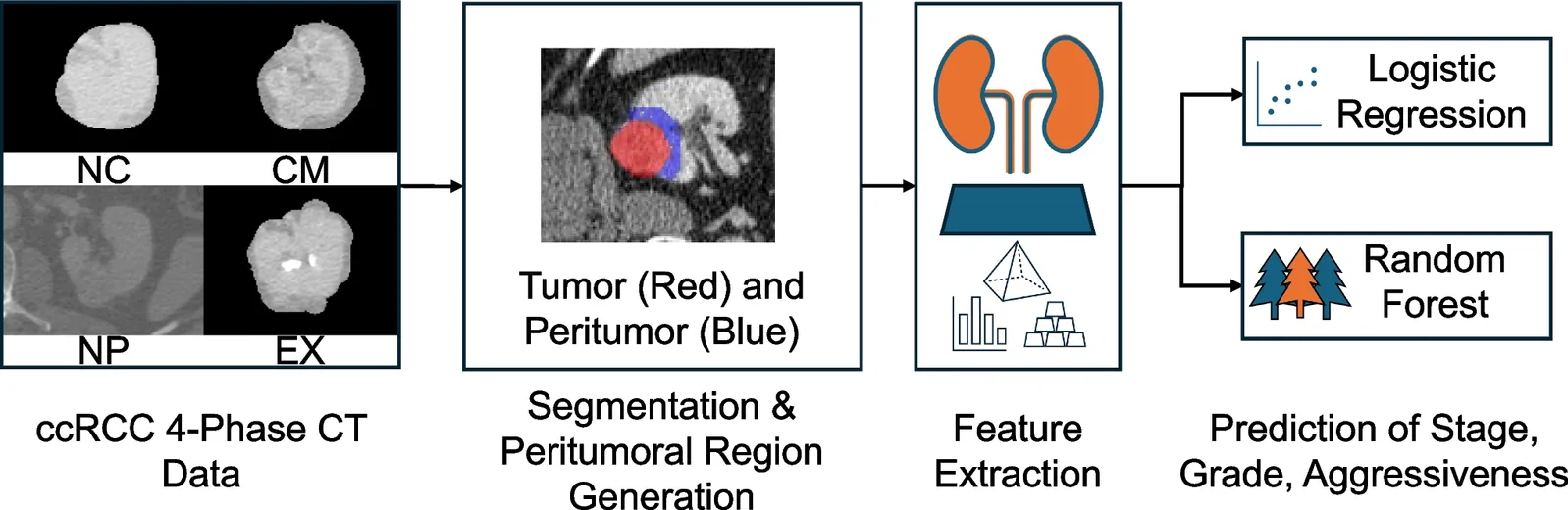
Workflow diagram for multiphasic CT-based radiomic analysis in ccRCC. Tumor and peritumoral regions were segmented, pre-processed, and used to extract radiomic features across four-phase CT. Features were used to train models for prediction of tumor grade, stage, and risk level, comparing tumor, peritumor, and combined models

This study aimed to develop and compare radiomic models for predicting ccRCC stage, grade, and aggressiveness. The core of our methodology was a systematic comparison of different feature spaces, which were defined by spatial (tumor, peritumoral, or combined) and temporal (single-phase or multiphase) factors. For each of the three clinical outcomes, we trained and evaluated multiple models to answer two key questions: (1) Do features derived from the peritumoral region improve prediction over tumor features alone? (2) Does using multiple CT phases improve prediction over using the best single phase? The study workflow is summarized in Fig. 1.

### Patients

This retrospective study was approved by the Institutional Review Board at the university-affiliated hospital. A total of 250 patients with pathologically confirmed clear cell renal cell carcinoma (ccRCC) were included in the study. All patients underwent four-phase contrast-enhanced CT within 1 year prior to nephrectomy at affiliate hospitals of the University of Southern California between 2009 and 2018. All CT images were obtained using a 64-detector row helical CT scanner. Exclusion criteria were (1) absence of any of the four CT phases (non-contrast, corticomedullary, nephrographic, or excretory), (2) absence of outcome data relating to tumor stage, grade, or aggressiveness, or (3) pathological diagnosis of a non-ccRCC tumor subtype.

### Segmentation and Image Processing

The primary tumor region of interest (ROI) was segmented in the nephrographic phase by multiple radiology residents under the direct supervision of a radiologist with over 20 years of experience in abdominal imaging. In cases of uncertain tumor borders, real-time consensus was obtained with the senior radiologist. This specific protocol has previously demonstrated a high degree of inter-observer reproducibility [10]. All other phases (non-contrast, corticomedullary, and excretory) were registered to this phase as a reference to ensure spatial alignment across phases.

**Fig. 2:**
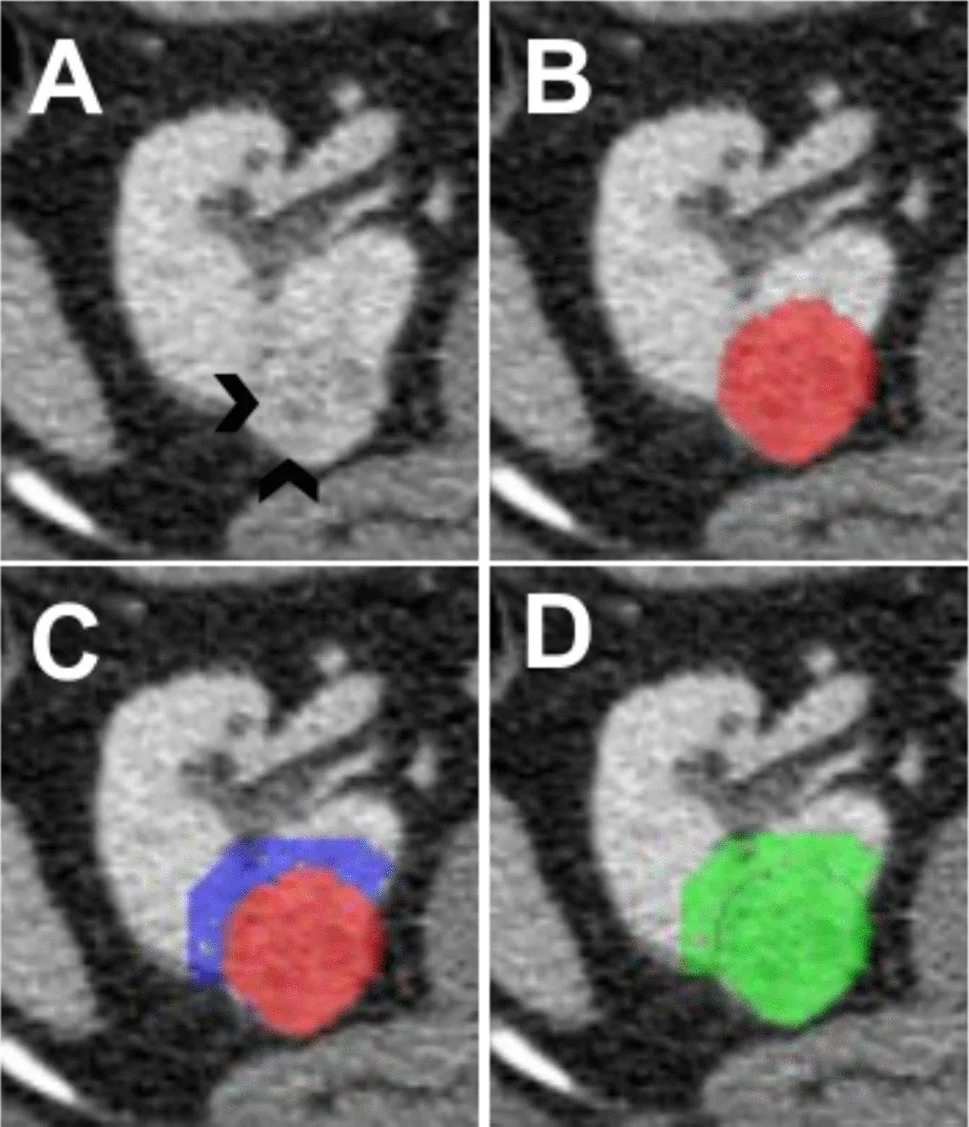
Extraction of the tumor and peritumoral regions of interest in a 60-year-old male with a partly exophytic right interpolar tumor (black arrowhead). **A** Tumor in renal parenchyma. **B** Tumor region highlighted in red. **C** Peritumoral region capturing 5-mm intraparenchymal shell. **D** Combined tumor and peritumoral regions

For each case, the four phases were identified: non-contrast (NC), corticomedullary (CM), nephrographic (NP), and excretory (EX). The tumor masks were pre-processed by filling any internal holes using a 3D binary morphological closing operation to ensure a contiguous tumor volume. A peritumoral region was generated around the resulting tumor mask by performing a 3D binary dilation to create a 5-mm shell surrounding the tumor. The peritumoral ROI was defined as the volume between this dilated mask and the original tumor mask (Fig. 2). Inclusion of perinephric fat, which lacks the relevant parenchymal microstructure, was minimized through exclusion of any peritumoral voxels with Hounsfield unit values below − 30 on the non-contrast phase, a threshold established in previous literature [15].

### Feature Extraction

Radiomic features were extracted from the tumor, peritumoral, and combined (tumor + peritumoral) regions for each of the four CT phases using a custom Python pipeline. All experiments were completed using Python 3.10.18 and PyRadiomics v.3.1.0 (https://pypi.org/project/pyradiomics/).

Inter-scanner variability was mitigated through multiple pre-processing harmonization steps prior to feature extraction. Images were resampled to an isotropic voxel spacing of 1 mm^3^ and voxel intensities were standardized using *z*-score normalization computed within each ROI to minimize the influence of absolute intensity differences. A fixed bin width of 25 Hounsfield units was used for texture feature calculation to guarantee a consistent intensity profile across all images. These normalization steps were applied independently for each region and imaging phase to ensure that the extracted texture features reflected local heterogeneity rather than systemic differences in scanners and scanner protocols.

A total of 1874 features were extracted per CT imaging phase for each region. The features extracted included shape (*n* = 14), first-order statistical features (*n* = 360), and texture features (*n* = 1500). The textural features included those from the Gray-Level Co-occurrence Matrix (*n* = 480), Gray-Level Dependence Matrix (*n* = 280), Gray-Level Run-Length Matrix (*n* = 320), Gray-Level Size Zone Matrix (*n* = 320), and Neighboring Gray-Tone Difference Matrix (*n* = 100).

### Feature Selection

Feature selection was performed to identify a robust and non-redundant feature set for prediction. Selection was performed independently within each cross-validation fold to prevent data leakage. For each training fold, we computed a Spearman correlation matrix and removed redundant features by excluding one feature from any pair with a correlation exceeding 0.90. The resulting feature subset was then applied to the corresponding test fold, resulting in median feature-to-sample ratios of multiphase models of 7.66–8.56:1 across all regions and outcomes (Supplementary Table 1). Single-phase models, which were trained on fewer features, demonstrated substantially lower ratios of 1.90–2.27:1.

Predictive models were developed using two supervised learning algorithms: Elastic Net logistic regression and Random Forest. These models were chosen for their complementary approaches to handling high-dimensional data. Elastic Net performs intrinsic feature selection through regularization and Random Forest evaluates feature importance through ensemble voting. For Elastic Net, we used the “saga” solver with a maximum of 10,000 iterations. For Random Forest, we used the scikit-learn implementation with default bootstrapping and Gini impurity for node splitting. Other hyperparameters for feature selection and classifier-specific parameters were optimized using a grid search (Supplementary Table 2).

### Statistical Analysis

Three binary outcomes were analyzed: tumor stage, tumor grade, and tumor aggressiveness. The tumor stage was binarized into low-stage ccRCC, comprised of pT1 tumors, and high-stage ccRCC, comprised of tumors of stage pT2a and above. Tumor grade was binarized into low-grade ccRCC, comprised of grade 1 and 2 tumors, and high-grade ccRCC, comprised of grade 3 and 4 tumors. Aggressiveness was binarized into aggressive and indolent lesions using histological classification as described by Bhindi et al., where aggressive tumors were those with histologic grade 3 or 4, any stage greater than 2, or evidence of sarcomatoid differentiation or necrosis [32].

Model performance was evaluated using tenfold stratified cross-validation, with the area under the receiver operating characteristic curve (AUC) as the primary metric. Stratification was used to preserve the proportion of each outcome class in every training and test fold, helping to mitigate bias from class imbalance. The final reported AUC was calculated by pooling all out-of-fold (OOF) predictions, providing a comprehensive estimate of model performance on unseen data. Confidence intervals for the AUC were computed using DeLong’s method. Secondary performance metrics included sensitivity, specificity, accuracy, F1-Score, and Brier Score. We trained and compared three distinct models for each outcome and algorithm: a tumor model (features from within the tumor ROI only), a peritumoral model (features from within the peritumoral ROI only), and a combined model (features from the fused tumor and peritumoral regions). Statistical comparisons between model AUCs were performed using DeLong’s test for two correlated receiver operating characteristic curves, with *p*-values < 0.05 considered to be statistically significant.

## Results

### Patient Clinical Characteristics

A total of 250 patients with ccRCC were included in the study. The cohort’s clinical and pathological characteristics are summarized in Table 1. The mean patient age was 60 years (SD 13), with a majority being male (72%). The mean tumor size was 3.86 cm (SD 2.18). The dataset exhibited class imbalance for the outcomes of interest: 70% of tumors were low-grade, 80% were low-stage (pT1), and 56% were classified as aggressive.

**Table 1:** Demographic details of patients with ccRCC

**Characteristics**		**Sample size**
Gender	Male	181 (72%)
	Female	69 (28%)
Age (mean, SD)		60 (13)
Size (mean, SD)		3.86 (2.18)
Outcome		
Low stage (pT1)		200 (80%)
High stage (pT2 +)		50 (20%)
Low grade (1 or 2)		175 (70%)
High grade (3 or 4)		75 (30%)
Non-aggressive		109 (44%)
Aggressive		141 (56%)

### Model Performance by Spatial Region

We first evaluated the predictive performance of models based on features from different spatial regions: the tumor, the peritumoral shell, and the composite combined area.

### Prediction of Tumor Stage

Models using intratumoral features achieved the highest performance on stage prediction (Table 2). The Elastic Net model trained on the tumor region yielded an AUC of 0.875 (95% CI = 0.822–0.928), significantly outperforming the combined model (AUC 0.828, *p* = 0.007). The peritumoral model demonstrated an intermediate AUC of 0.838. All stage prediction models were characterized by very high specificity (> 0.94) but modest sensitivity (0.34–0.44), indicating a superior ability to correctly identify low-stage tumors compared to the detection of higher-stage disease. Random Forest classifiers consistently underperformed relative to Elastic Net across all regions for this outcome.

**Table 2:** Performance metrics for the prediction of ccRCC stage

Elastic Net model						
Region	OOF AUC (95% CI)	Sensitivity	Specificity	Accuracy	F1-Score	Brier Score
Tumor	0.875 (0.822–0.928)	0.400	0.985	0.868	0.548	0.100
Peritumor	0.838 (0.773–0.903)	0.340	0.990	0.860	0.493	0.110
Combined	0.828 (0.765–0.890)	0.400	0.945	0.836	0.494	0.115
Random Forest model						
Region	OOF AUC (95% CI)	Sensitivity	Specificity	Accuracy	F1-Score	Brier Score
Tumor	0.832 (0.763–0.900)	0.440	0.960	0.856	0.550	0.109
Peritumor	0.796 (0.715–0.877)	0.420	0.965	0.856	0.538	0.112
Combined	0.804 (0.726–0.882)	0.400	0.980	0.864	0.541	0.109

### Prediction of Tumor Grade

Model performance was lower for grade prediction than for stage across all regions (Table 3). Consistent with the stage results, the optimal model was an Elastic Net classifier trained on tumor-region features (AUC 0.697, 95% CI = 0.628–0.765). The combined model performed poorly (AUC 0.626), and the difference from the tumor-only model was not statistically significant (*p* = 0.108). Notably, the sensitivity of the best-performing model was low (0.147), suggesting further difficulty in correctly identifying high-grade tumors. The higher Brier scores across all models (0.194–0.281) reflected poorer probability calibration for grade compared to other outcomes.

**Table 3:** Performance metrics for the prediction of ccRCC grade

Elastic Net model						
Region	OOF AUC (95% CI)	Sensitivity	Specificity	Accuracy	F1-Score	Brier Score
Tumor	0.697 (0.628–0.765)	0.147	0.943	0.704	0.229	0.194
Peritumor	0.621 (0.546–0.696)	0.200	0.909	0.696	0.283	0.207
Combined	0.626 (0.552–0.701)	0.413	0.743	0.644	0.411	0.281
Random Forest model						
Region	OOF AUC (95% CI)	Sensitivity	Specificity	Accuracy	F1-Score	Brier Score
Tumor	0.612 (0.536–0.687)	0.093	0.954	0.696	0.156	0.204
Peritumor	0.643 (0.567–0.719)	0.147	0.954	0.712	0.234	0.199
Combined	0.603 (0.525–0.681)	0.093	0.931	0.680	0.149	0.207

### Prediction of Tumor Aggressiveness

All models demonstrated strong performance in the prediction of the composite outcome of tumor aggressiveness (Table 4). The tumor-region model again achieved the highest AUC of 0.915 (95% CI = 0.881–0.949) using the Elastic Net classifier, significantly outperforming both the peritumoral (*p* = 0.009) and combined (*p* = 0.004) models. In contrast to stage and grade predictions, the aggressiveness models achieved comparatively balanced performance, with high sensitivity (0.82–0.89) and moderate specificity (0.71–0.76).

**Table 4:** Performance metrics for the prediction of ccRCC aggressiveness

Elastic Net model						
Region	OOF AUC (95% CI)	Sensitivity	Specificity	Accuracy	F1-Score	Brier Score
Tumor	0.915 (0.881–0.949)	0.894	0.725	0.820	0.848	0.128
Peritumor	0.869 (0.827–0.912)	0.837	0.697	0.712	0.808	0.156
Combined	0.881 (0.840–0.921)	0.823	0.716	0.776	0.806	0.142
Random Forest model						
Region	OOF AUC (95% CI)	Sensitivity	Specificity	Accuracy	F1-Score	Brier Score
Tumor	0.911 (0.876–0.945)	0.865	0.752	0.816	0.841	0.125
Peritumor	0.860 (0.816–0.904)	0.816	0.706	0.768	0.799	0.156
Combined	0.886 (0.846–0.926)	0.830	0.761	0.800	0.824	0.140

### Single-Phase and Multiphase Analysis

**Fig. 3:**
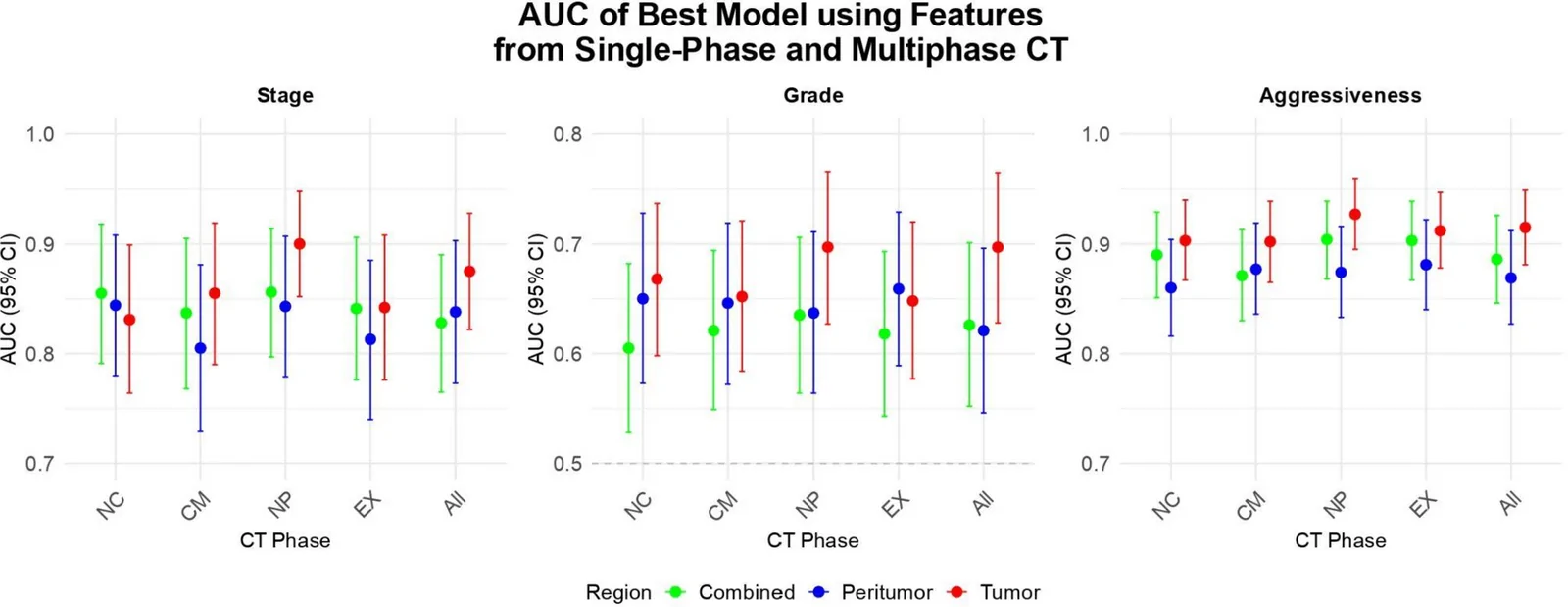
Comparison of the mean AUC of the best-performing single-phase and multiphase models across stage, grade, and aggressiveness outcomes

We further investigated whether leveraging multiple CT phases improved predictive performance compared to using only the best single phase. The results, summarized in Fig. 3 and detailed in Supplementary Table 3, demonstrated that multiphase analysis provided no meaningful benefit. For nearly all regions and outcomes, the best single-phase model matched or outperformed the best multiphase model. The sole exception was in grade prediction using the tumor region, where multiphase features provided equivalent performance to the best single-phase model.

Critically, an Elastic Net model using only nephrographic-phase intratumoral features demonstrated the best overall performance, showing equal or superior predictive ability to the best multiphase models for stage (AUC 0.900 vs. 0.875, *p* = 0.493), grade (AUC 0.697 vs. 0.697, *p* = 0.994), and aggressiveness (AUC 0.927 vs. 0.915, *p* = 0.522). Within the nephrographic phase, texture features made up the majority of the most influential predictors for all three outcomes, representing 55–65% of the top 20 features. First-order features were the second most contributory class (15–25%).

## Discussion

In this retrospective study, we conducted a comprehensive evaluation of multiphase CT-based radiomics for prediction of stage, grade, and aggressiveness in ccRCC. Our comparison yielded two principal findings. First, contrary to our hypothesis, incorporating peritumoral features did not improve predictive performance. Models based solely on the tumor region consistently achieve superior results. Second, we found that a simplified model using only the nephrographic phase outperformed more complex models leveraging the full multiphasic protocol. Together, these results suggest that, for the examined tasks, the most informative signals are captured within the primary tumor during the nephrographic phase.

The failure of the peritumoral region to add predictive value stands in contrast to prior work by Zhou et al. and Li et al., who reported significant improvements in grade prediction from inclusion of the peritumoral region in analysis [27, 28]. The divergence in results may be attributed to several methodological differences. Our study employed a highly conservative feature selection protocol, rigorously guarding against data leakage, which may have more stringently filtered out less robust peritumoral features [33]. Furthermore, our peritumoral segmentation strategy, which used a fixed 5-mm shell and actively excluded perinephric fat, was intentionally designed to capture intraparenchymal changes specific to the tumor interface. It is possible that alternative segmentation approaches, such as those that accommodate smaller margins or a fat-inclusive perinephric region, encode different information of biological relevance. It is worth noting that the performance of our tumor-region model for grade prediction (AUC 0.697) aligns closely with the tumor-region models in Li et al.’s study (AUC 0.66–0.70) [28]. This consistency suggests that, while textural differences in the peritumor environment exist, the core predictive signal resides within the tumor, and the reported benefit of the peritumoral region may be contingent on cohort characteristics and analytical choices, rather than being a universal phenomenon [34].

Similarly, the lack of benefit from multiphase analysis is a critical finding with direct implications for research efficiency. The nephrographic phase, which best depicts renal parenchymal enhancement and tumor contrast, provided all necessary information for prediction of the studied outcomes. The non-contrast, corticomedullary, and excretory phases did not contribute complementary data that improved model performance. While we originally posited that these phases would offer additional predictive utility due to their exposure to angiogenesis and washout effects, the data shows that the textural and first-order features most predictive of tumor biology are best captured in this single, standard phase, rendering the additional computational burden of multiphase analysis unnecessary.

Our study has several limitations. The retrospective, single-center design and cohort size may affect the generalizability of our findings, particularly in the absence of external validation using multi-institutional data or data from a wide range of scanners. Potential scanner heterogeneity effects were not explicitly addressed, which could impact model performance across diverse imaging platforms. The dataset was characterized by class imbalance, with a substantially greater portion of the study population demonstrating mild outcomes (low-stage and low-grade) than severe outcomes. While we employed stratified cross-validation to address these class imbalances, the low prevalence of high-stage and high-grade tumors may limit sensitivity for these minority classes. Our models also did not incorporate clinical variables, such as patient demographics, which may provide complementary predictive information to future research. Furthermore, our outcome groupings, such as grade 3 and 4 tumors being categorized as “high-grade,” may oversimplify nuanced prognostic information. Finally, manual segmentation introduces subjectivity and may limit reproducibility.

This study demonstrates that, for our cohort, nephrographic-phase radiomics involving the tumor region provides optimal predictive performance for stage, grade, and aggressiveness in ccRCC. Further studies should pursue prospective, multi-institutional validation to confirm the generalizability of these findings. The incorporation of automated segmentation methods may reduce subjectivity and lay the groundwork for an end-to-end autonomous prediction tool, which could increase utility in the clinical setting. Additionally, the integration of complementary data streams, including genomic and pathology-based biomarkers, could further enhance the precision of predictive models and personalized risk stratification. By advancing these directions, future research may accelerate the translation of radiomic tools into clinical workflows, ultimately providing interpretable models that clinicians may use to inform patient care.

## Conclusion

This study demonstrates that a simplified radiomic approach using features from the tumor region in the nephrographic phase of CT alone yields optimal performance in the prediction of ccRCC stage, grade, and aggressiveness. Contrary to our initial hypothesis, the use of peritumoral features and multiphase analysis did not enhance predictive accuracy, often instead leading to poorer model performance. These findings provide practical guidance for future research, suggesting further studies may focus on refining single-phase, tumor-based models rather than incorporating more complicated multiphase and multiregional feature spaces. Future work should focus on multi-institutional validation and the integration of clinical and molecular data to build clinically translatable tools for risk stratification.

## Supplementary Information

Below is the link to the electronic supplementary material.

Supplementary Material 1 (DOCX 20.4 KB)

## Data Availability

Deidentified data may be available per university IRB rules and regulations for private review access.
