# Supplementary material for: Multiphase CT-Based Tumor and Peritumoral Radiomics for Characterization of Clear Cell Renal Cell Carcinoma
Source: J Imaging Inform Med. 2026 Jan 5;39(5):4115–23. doi: 10.1007/s10278-025-01797-9 (PMC13694603; doi:10.1007/s10278-025-01797-9)
Supplement: Supplementary file 1 [file 10278_2025_1797_MOESM1_ESM.docx]

**Supplementary Table 1:** Feature dimensionality following within-fold correlation filtering (Spearman’s ρ > 0.9) for each region and outcome in multiphase analysis.

| Region | Outcome | Median Feature-to-Sample Ratio |
| --- | --- | --- |
| Tumor | Stage | 7.69:1 |
|  | Grade | 7.66:1 |
|  | Aggressiveness | 7.67:1 |
| Peritumor | Stage | 8.56:1 |
|  | Grade | 8.55:1 |
|  | Aggressiveness | 8.56:1 |
| Combined | Stage | 8.25:1 |
|  | Grade | 8.24:1 |
|  | Aggressiveness | 8.25:1 |

**Supplementary Table 2:** Hyperparameter values used for model tuning.

| Model | Fixed Parameters | Hyperparameter | Tested Values |
| --- | --- | --- | --- |
| Elastic Net  Logistic Regression | solver = ‘saga’,  max_iter = 10000,  penalty = ‘elasticnet’ | C | 0.01, 0.1, 1 |
|  |  | l1_ratio | 0.1, 0.5, 0.9 |
| Random Forest | bootstrap = True  criterion = ‘gini’ | Number of Estimators | 100 |
|  |  | Max Depth | 3, 5, 10 |
|  |  | Minimum Samples in Leaf Node | 5 |
|  |  | Max Features | Sqrt |

**Supplementary Table 3:** AUC values (95% confidence intervals) for models using features from single-phase and multiphase CT across each region and outcome.

| Region | Outcome | NC Phase | CM Phase | NP Phase | EX Phase | Multiphase |
| --- | --- | --- | --- | --- | --- | --- |
| Tumor | Stage | 0.831 (0.764-0.899) | 0.855 (0.790-0.919) | 0.900 (0.852-0.948) | 0.842 (0.776-0.908) | 0.875 (0.822-0.928) |
|  | Grade | 0.668 (0.598-0.737) | 0.652 (0.584-0.721) | 0.697 (0.627-0.766) | 0.648 (0.577-0.720) | 0.697 (0.628-0.765) |
|  | Aggressiveness | 0.903 (0.867-0.940) | 0.902 (0.865-0.939) | 0.927 (0.895-0.959) | 0.912 (0.878-0.947) | 0.915 (0.881-0.949) |
| Peritumor | Stage | 0.844 (0.780-0.908) | 0.805 (0.729-0.881) | 0.843 (0.779-0.907) | 0.813 (0.740-0.885) | 0.838 (0.773-0.903) |
|  | Grade | 0.650 (0.573-0.728) | 0.646 (0.572-0.719) | 0.637 (0.564-0.711) | 0.659 (0.589-0.729) | 0.621 (0.546-0.696) |
|  | Aggressiveness | 0.860 (0.816-0.904) | 0.877 (0.836-0.919) | 0.874 (0.833-0.916) | 0.881 (0.840-0.922) | 0.869 (0.827-0.912) |
| Combined | Stage | 0.855 (0.791-0.918) | 0.837 (0.768-0.905) | 0.856 (0.797-0.914) | 0.841 (0.776-0.906) | 0.828 (0.765-0.890) |
|  | Grade | 0.605 (0.528-0.682) | 0.621 (0.549-0.694) | 0.635 (0.564-0.706) | 0.618 (0.543-0.693) | 0.626 (0.552-0.701) |
|  | Aggressiveness | 0.890 (0.851-0.929) | 0.871 (0.830-0.913) | 0.904 (0.868-0.939) | 0.903 (0.867-0.939) | 0.886 (0.846-0.926) |
